# INDOOR AND OUTDOOR FALLS AMONG ADULTS AGING WITH A PHYSICAL DISABILITY

**DOI:** 10.1093/geroni/igz038.448

**Published:** 2019-11-08

**Authors:** Thomas J Eagen, Tracy Mroz, Tracy Chippendale, Carrie Karvonen-Gutierrez, Sam Battalio, Katie Singsank, Ivan Molton

**Affiliations:** 1 University of Washington, Seattle, Washington, United States; 2 New York University, New York, New York, United States; 3 University of Michigan, Ann Arbor, Michigan, United States

## Abstract

Falls among community-dwelling adults are a significant public health concern. Adults aging with a physical disability report a high number of falls, recurrent falls, and injuries caused by falls. Prevention strategies are needed to reduce the incidence of falls among this population; however, the location of the fall may influence which strategies will be most effective. The purpose of this project was to examine falling indoors versus outdoors was associated with fall related psychological concerns (e.g., self-efficacy), self-reported physical activity levels, physical function and sociodemographic characteristics (e.g., sex, age, education, employment), using survey data of adults aging with four conditions: muscular dystrophy (MD), multiple sclerosis (MS), post-polio syndrome (PPS), and spinal cord injury (SCI). Of the 1381 participants who completed the survey in 2017, 52% (n=719) reported at least one fall in the past 6 months. When asked about their worst fall, 32% of falls (n=233) occurred outdoors and 68% (n=486) occurred indoors. Participants with MS were significantly more likely to report falling outdoors (MS=35%, MD = 21%, PPS = 21%, SCI = 24%). Factors significantly associated with outdoor falls included living in an urban environment (OR = 1.59; 95%CI:1.06, 2.39), being more physically active (OR = 1.01; 95%CI:1.001, 1.02) and having better physical function (OR = 1.05; 95%CI:1.03, 1.08). These results fill a critical gap in the falls literature on fall location and have important implications for tailoring fall prevention interventions for individuals aging with a physical disability.

